# A Qualitative Study Exploring the Experiences of Mothers Caring for Their Children with Thalassemia in Iran

**DOI:** 10.4314/ejhs.v31i6.25

**Published:** 2021-11

**Authors:** Majedeh Nabavian, Fatemeh Cheraghi, Farshid Shamsaei, Lily Tapak, Ahmad Tamadoni

**Affiliations:** 1 Nursing and Midwifery School, Hamadan University of Medical Sciences, Hamadan, Iran; 2 Chronic Diseases (Home Care) Research Center, Hamadan University of Medical Sciences, School of Nursing and Midwifery, Hamadan, Iran; 3 Maternal and Child Care Research Center, Behavioral Disorders and Substance Abuse Research Center, Hamadan University of Medical Sciences, Hamadan, Iran; 4 Department of Biostatistics, School of Public Health, Modeling of Noncommunicable Diseases Research Center, Hamadan University of Medical Sciences, Hamadan, Iran; 5 Non-Communicable Pediatric Disease Research Center, Health Research Institute, Babol University of Medical Sciences, Babol, Iran

**Keywords:** beta-Thalassemia, Mothers, Parents, Child Care, Qualitative Study

## Abstract

**Background:**

Mothers of children with thalassemia usually experience many sufferings and challenges in caring of their children. The present study aimed to explore the experiences of mothers caring for their children with thalassemia.

**Methods:**

In this qualitative study, 14 mothers caring for their children with thalassemia in Hamedan and Babol Cities, Iran were selected using purposeful sampling, from December 2019 to August 2020. Data were collected through semi-structured face-to-face interviews. Graneheim and Lundman's approach of conventional content analysis was used for data analysis.

**Results:**

After data analyzing, four themes, including physical distress, psychological suffering, hellish life, and self-negligence, as well as nine categories, including the mother's physical problems, physical weakness, confusion, painful emotions, restless life, involvement in a painful caring process, turmoil in the family, neglect of one's health, and disregard for the occurrence of psychosomatic illnesses, were extracted.

**Conclusion:**

Our findings provide a broad range of context-specific challenges that mothers of thalassemic child faced during caring of their children that can affect different aspects of their life and health. Thus, mothers of children with thalassemia need various types of support such as social, emotional, and informational support during caring process of their children.

## Introduction

Thalassemia is a genetic and chronic congenital anemia with minor and major types (1). Thalassemia major caused by hemoglobinopathies, is the most common genetic disease in humans and is more prevalent (2.5–15%) in the Mediterranean and tropical areas (2). About 240 million people in the world are carriers of beta-thalassemia, and about 200000 thalassemia major patients exist worldwide, with about 60000 thalassemia major patients being added to this number annually. Iran has about 3 million carriers of thalassemia, which is significantly (5.1%) higher than the global average (1). About 25000 thalassemia major patients live in Iran, and about 800 thalassemic infants are born annually in the country (1–2). As a severe disease, the long-term prognosis of thalassemia major depends on the treatment adherence to transfusion and iron chelation therapies. Also, like other patients with chronic diseases, these children need lifelong treatment and face many physical, psychological, and social problems (3). This disease has significant impacts on the growth and development process in the first year of life. The physical development of a thalassemic child is lower than that of their peers, which may result in their poor self-care management and quality of life (1). Frequent referrals of these patients to medical centers for blood transfusion lead to a changes in their lifestyle, school absenteeism, reduced social activities, and social isolation (4). Additionally, it has been previously revealed a high prevalence of cardiac and endocrine complications in children suffering from thalassemia major (5). Since thalassemia is a genetic disease that begins in childhood, the mother plays a crucial role in caring process of their thalassemic children. Studies have shown that mothers play an active role in caring for their children and spend much time with them (6, 7). Moreover, in Iranian culture and society, mothers are assumed to have the most important role in caring for a sick child (7–8).

These mothers are usually involved in caring process of the child during long periods of treatment, which cause major physical, social and financial burdens on them (6, 8). It has been previously indicated that disabled children's mothers have lower physical and psychological health (6, 8–9). Mothers of thalassemic Childs usually face a variety of tensions which can have negative effects on their life (7). The results of a study indicated that about 1.5% of thalassemic children's mothers in the first two years of their children's treatment usually suffered from emotional disorders and various physical diseases (10). Also, playing multiple roles in caring for thalassemic children and worrying about their future caused enormous psychological stress to the mothers of thalassemic children (11). Transmitting the thalassemia gene to the child and being responsible for the child's disease also cause feeling of guilt and self-blamein the mother (12, 13). Pouraboli et al. in their study showed that mothers are reluctant to reveal the disease in their children due to the stigma attached to thalassemia, which consequently causes physical exhaustion, stress, and deprivation of others' support (14).

Mothers' experiences of caring for a thalassemic child are associated with several challenges influenced by various variables and factors. The conventional content analysis approach can reveal the hidden aspects of such experiences. Mothers have the main role in caring for an ill child. Their experiences in caring for the patient and their perception about the disease can be evaluated using conventional content analysis (15–17). There is a dearth of qualitative research worldwide on these mothers' experiences, especially in Iran. Qualitative studies can provide a deeper understanding of challenges usually experienced by mothers with thalassemia children. Therefore, the present study aimed to explore the experiences of mothers caring for their children with thalassemia.

## Methods

**Study design and participants**: This study was conducted using qualitative content analysis with a conventional approach. Qualitative content analysis is a research method used to interpret textual data through systematic classification, coding, creating themes, and designing known models (18–19). In this study, 14 mothers of thalassemic children were selected by purposeful sampling method from two thalassemia centers in Babol and Hamedan city, Iran, from December 2019 to August 2020. The inclusion criteria were having a child with thalassemia major, the mother as the primary caregiver, having both parents, and the absence of chronic psychiatric disorders in the mother.

An agreement on data saturation was reached with the participation of 14 individuals. After 12 interviews, data saturation was reached as no further theme was obtained. However, the research team conducted two more interviews to confirm the absence of new themes.

**Data collection**: Semi-structured in-depth interviews were used based on the guidelines for interviewing to collect data. A private room in the hospital was used for the interviews. Interviews were performed from December 2019 to August 2020. The interview began with open-ended questions like “*How has caring for a thalassemic child affected your life?*” and followed by questions like “*Tell me about your experience of caring for a thalassemic child*.” The interviews lasted from 30 to 60 minutes, depending on the participants' tolerance and interests. One of the researchers performed and audio recorded all the interviews. The researcher carefully listened to the interview at the end of each session and transcribed the audio verbatim. The interviewer also recorded the participants' body language and expressions.

**Data analysis:** The data were analyzed using of five steps conventional content analysis approach according to the technique described by Graneheim and Lundman's (20). Immediately after each interview, it was transcribed word by word and the transcript was reviewed to grasp its main ideas. Then, each interview transcript was considered as the unit of analysis and meaning units were identified and coded. According to their similarities, generated codes were grouped into subcategories. Finally, subcategories were compared and grouped to develop larger categories and identify the latent content of the data.

**Ethical considerations**: The ethics committee of the Hamadan University of Medical Sciences approved the study (IR.UMSHA.REC. 1399.167). An informed consent document provided to each participant explained the research's purpose and detailed potential risks. The document also provided a confidentiality statement of how participant information would be securely handled.

**Rigor of the study**: Lincoln and Guba's criteria for creditability, transferability, dependability, and confirmability were evaluated to ensure that the study's rigor was achieved (21, 22). Credibility was addressed by recruiting diverse individuals by considering age, the difference in duration of the conflict with experience, and different hospital units. Transferability was achieved by in-depth descriptions of the mothers' experiences sampled from two hospitals in different cities. Dependability was ensured by closely following data analysis procedures of the in-depth descriptions and transcription of mothers' experiences. Lastly, confirmability was achieved by eliminating the research team's biases (to maintain openness to the participants' information).

## Results

Fourteen mothers of thalassemic children participated in the study (Table 1).

**Table 1:** Demographic characteristics of the participants

Participants	Age	Employed	Education	Consanguinity between parents	Number of thalassemic children
Participant 1	31	yes	High school	yes	More than one child
Participant 2	34	no	diploma	yes	One child
Participant 3	43	no	High school	no	One child
Participant 4	45	no	High school	no	One child
Participant 5	35	no	High school	no	One child
Participant 6	38	no	High school	yes	One child
Participant 7	33	yes	diploma	no	One child
Participant 8	38	no	diploma	no	More than one child
Participant 9	28	yes	university	yes	One child
Participant 10	22	no	High school	no	One child
Participant 11	50	yes	University	no	One child
Participant 12	40	no	High school	yes	More than one child
Participant 13	25	no	High school	no	One child
Participant 14	37	yes	university	yes	More than one child

The analysis of the participants' experiences yielded four themes, including “physical distress”, “psychological suffering”, “hellish life”, “self-negligence”, and nine categories, including the “mother's physical problems”, “physical weakness”, “confusion”, “painful emotions”, “restless life”, “involvement in a painful caring process”, “turmoil in the family”, “neglect of one's health”, and “disregard for the occurrence of psychosomatic illnesses” (Table 2).

**Table 2:** Themes and categories extracted from the mothers' experiences

Categories	Themes
Maternal physical problems	Physical distress
Physical weakness and exhaustion	
Confusion	Psychological suffering
Painful emotions	
Restless life	Hellish life
Involvement in the bitter process of caring	
Turmoil in the family	
Disregarding the occurrence of psychosomatic illnesses	Self-negligence
Neglecting one's health	

**Physical distress**: The participants reported various acute and chronic physical problems in their bodily organs and systems. They mostly reported that these problems were due to injuries, harms, stress, weakness, and fatigue caused by the long-term care of their ill children. This theme includes the following two categories:

**A) Maternal physical problems**: The occurrence of physical discomfort and pains in different bodily systems was an experience of many of the participants who felt that they lost their physical health.

They stated that their physical problems were due to the grief of having ill children and taking continuous care of them. These mothers stated a wide range of acute and chronic diseases as their experiences. One of the participants (No. 3) stated in this regard: “*When I received the news of my illness, my heart sank. I now have a headache, stomach ache and heart disease. Sometimes I am so upset that as if acid has been poured into my stomach. It is so difficult to take care of an ill child that my hands hurt. I can no longer do house works*.”

**B) Physical weakness and exhaustion:** The participants felt weak and lost their physical strength, and thus, they could no longer perform their affairs and provide care for their ill children. The mothers stated that they became physically weak and exhausted due to the fatigue caused by caring for their ill children, and the energy loss prevented them from doing anything. “*I feel a strange tiredness that has made me weak, I was very active before but not now. My hands and feet are falling asleep and I do not have any motivation to do something. I'm trying to be resilient, but I am slowly losing my energy*” (Participant No. 7).2.

**Psychological suffering:** The mothers' experiences showed that their mental health was gradually challenged. In describing their experiences, they stated that their morale was weakened, they were neurologically intolerant, and they were affected psychologically. In this regard, two categories were extracted, including confusion and painful emotions.

***A) Confusion***: All the participants experienced persistent feelings of worry and anxiety. Most of them felt sadness and restlessness and had disturbed thoughts that threatened their health. Participant No. 12 expressed her mental health condition: “*Now I'm not calm anymore, sometimes I say I'm sick, I'm not really nervous. My heart is always full of chaos and turmoil*.”
***B) Painful emotions:*** Many of the participants experienced inner pain and suffering and became emotionally vulnerable by losing their peace of mind, which is often described as depression and is usually associated with feelings of loss and lack of motivation. In this regard, Participant No. 5 stated, “*Sometimes I get so depressed that my daughter tells me, Mom, why are you like this? I always think about my daughter's illness. My daughter illness is a great suffering. I suffer so much that I want to die. I am a mother and I feel miserable due to her illness*”.

**Hellish life:** Caring for a thalassemic child creates many challenges for the mother and thus disrupts their lives and gradually reduces their health level. In this study, the above experience is described with three categories of “restless life,” “involvement in a painful caring process,” and “turmoil in the family”.

***A) Restless life***: Having a hard life, feeling hopeless about life, having stress in relationships with others, and worrying about the future of ill children were some of the life-challenging experiences of the thalassemic children's mothers. Participant No. 5 says in this regard: “*I do not have peace in my life, I say every day that now something bad happens to my child, only God knows how hard my life has become, my child has several problems, I suffer high pressure, and I bear them alone*.”

***B) Involvement with a painful caring process***: Thalassemic children need continuous treatment and care after being discharged from medical centers, and mothers often take on this responsibility alone. Playing the role of caregiver endangers the mother's health in the long run.

One of the participants' experiences in this study was difficulties and hardships caused by the long-term care of ill children manifested in experiences such as interruption in performing tasks, feeling of exhaustion and confusion, and enduring financial pressure. Participant No.2 said, “*I always have to put all my works aside and just take care of my son. I have not had a good sleep since my son affected by thalassemia. I have always been upset and crying because of hardships and pressures I have*.”

**C) Turmoil in the family**: The thalassemic child changes the emotional atmosphere in the family and the relationship between family members, and as the pressure increases, the mother becomes more vulnerable. The participants' experiences in describing this category included tension in the family atmosphere, disruption of emotional relationships among family members, reduced interactions within the family, and chaos in the home environment that could damage their health status. Participant No. 9 stated: “*My daughter's illness has made her siblings nervous so that when something happens, other children and their father get angry and shout, no one laughs at home anymore, when one in the house is bored, I just have to endure everything. If my husband helped me, I would be better*”.

**Self-negligence:** Despite physical problems in their bodily organs and systems, the participants paid less attention to their health. In this regard, the two categories included disregarding the occurrence of psychosomatic illnesses and not paying attention to health.

**A) Disregarding the occurrence of psychosomatic illnesses**: Many of the participants experienced pain and physical discomfort in their bodily systems. They felt that they had lost their physical health and considered high-stress tolerance a critical factor in the occurrence of physical symptoms. In other words, loss of happiness and the occurrence of anxiety and psychological stress were associated with the creation and exacerbation of physical problems and led to the occurrence and intensification of psycho-physical reactions in them. In this regard, Participants No. 6 stated, “*I am upset, and sadness affects all parts of my body, for example, I had a pain in my chest and throat yesterday. These pains are not important for me since, all my thought is on my daughter, I endure my pains*”.

**B) Neglecting of one's health**: although the physical health of mothers responsible for caring for a thalassemic child is at risk, they do not show much care in following up and treating it. In this regard, Participant No. 9 mentioned, “*I do not control my blood sugar because I require money or I do not go to physician for my high blood pressure, leave it, my children are more important and obligatory*.”

## Discussion

The results of this study are a good indication of suffering experienced by a thalassemic child's mother (23–24). Some studies have reported challenges of these mothers caused by the disease and their painful lives, which pose a significant risk to their health. In the present study, one of the most important challenges for the mothers was their physical distress. Other studies have reported that mothers experience a variety of physical problems such as fatigue, weakness, acute and chronic diseases (8, 24). Dahnil et al., showed that thalassemic children's parents, especially mothers, suffered more from physical and social problems than their children and needed comprehensive support for caring for their children (25). A study conducted by Abu Shusha has shown that thalassemic children's mothers suffer from many physical problems (26). Also, it has been shown that thalassemic children's parents experienced many physical and family problems (24). Relatives and the healthcare providers should take special measures to support these mothers to solve these problems and continue the care process. It is also necessary for healthcare providers to provide necessary support to mothers of patients with thalassemia. In this regard, it is essential to run counseling programs and refer these mothers to the related centers to enhance their self-care knowledge.

In the present study, another critical challenge faced by mothers of thalassemic children was psychological suffering. Other studies have reported that mothers experience suffering such as extreme fatigue and psychological complications (7, 24). A study conducted by Abu Shusha showed that thalassemic children's mothers suffered from many psychological problems (26). Widayanti et al. revealed that thalassemia is a genetic disorder and inherited from a patient's parents, mothers of children of thalassemia experience severe emotional conflicts such as feelings of guilt in the mother (27). Therefore, some measures should be taken by providing care facilities to alleviate these mothers' sufferings. In this regard, psychologists and psychiatric nurses have a critical role in identifying and treating, and supporting mothers of thalassemic children with psychological problems. In this study, one of the mothers' challenging experiences was having a hellish life caused by significant concerns, economic problems and chaos, and the family's turmoil that reduced their health level. The mothers were anxious about their children's future education, work, and marriage, which caused turmoil in their lives.

In a study conducted by Prasomsuk et al., mothers were worried about their children's future job (28). In Wahab's study, mothers were also worried about their children's independence and future jobs (29). Participants in the study carried out by Widayanti et al. were also worried about their children's future (27). In the study conducted by Lim et al., parents were also worried and frustrated about the current and future situation of their children (30). Healthcare providers' should provide mothers with appropriate information about the disease and strategies to provide care for their thalassemic children. The marriage of these children was also among the mothers' concerns. In studies which conducted in other countries, this worry was not reported, indicating the importance of marriage in Islamic-Iranian culture.

Another problem of the mothers was their poor financial situation, which caused a challenge for them in the painful caring process. It has been previously shown that families of children with chronic disease requiring long-term treatment, usually faced with major economic challenges (29–30). Therefore, governments should provide economic support to these families and provide drugs for their children. Also, by establishing private institutions with the participation of the public sector without imposing economic costs on families, it is possible to reduce the economic challenges of these mothers. Another challenges of the mothers was the family's emotional turmoil, which increased their vulnerability and intensified their pressures. In this regard, the study conducted by Abedi et al. showed that stress of thalassemic child can affect the whole family and causes emotional stress within the family (31). Madmoli et al., in their study, reported that thalassemia caused mental pressure within the family (32). Measures should be taken to establish special centers that provide necessary counseling programs to support these families and educate them on how to provide self-caring. The cooperation of psychiatric nurses and clinical psychologists in thalassemia care centers is also useful in managing the problems of these families.

In the present study, the mothers often neglected their own disease and did not take appropriate action on their disease, due to the great sufferings of child care. The mothers believed that they did not have enough time to take care of their health and that psychological stress reduced their physical strength. Previous studies revealed that mothers of a child with a chronic diseases usually ignored their essential needs, due to excessive responsibilities in caring for their children (33). Saldanhasj et al. showed that 1.5% of mothers of thalassemic children in the first two years of treatment of their child suffer from psychosomatic disorders and need others' support (34). Dahnil et al. found in their study that parents of thalassemic children, especially mothers, suffered from more physical and mental problems compared to their ill children and needed comprehensive support (25). The family and the healthcare providers should support these mothers to resolve this issue. In this regard, the government should prepare a strategic thalassemia program and train a thalassemia specialist team in universities to teach self-care and support these mothers.

Recall bias from study participants during the data collection and lack of generalizability of the findings to other mothers with thalassemic children in different geo-cultural contexts, due to small sample size and participants characteristics are the limitations of this study.

In conclusion, the experiences of mothers with thalassemic children in caring of their children revealed that they have many challenges in different dimensions in caring of their children. Therefore, providing comprehensive and appropriate health services to these mothers and ensuring psychiatric nurses' cooperation in solving mothers' psychological problems are beneficial. Also, enhancing mothers' awareness of self-care and the ways of providing care for ill children, implementing periodic educational programs for other family members and close relatives of thalassemia patients, establishing private institutions with the participation of the public sector without imposing economic costs on families, and enhancing public awareness about thalassemia through media are necessary.
